# Monitoring of Volatile Additives from Plant Protection Products in Tomatoes Using HS-SPME-GC-HRMS: Targeted and Suspect Approaches

**DOI:** 10.1021/acs.jafc.3c03280

**Published:** 2023-09-21

**Authors:** Jesús Marín-Sáez, Rosalía López-Ruiz, Roberto Romero-Gonzalez, Antonia Garrido Frenich

**Affiliations:** † Research Group “Analytical Chemistry of Contaminants”, Department of Chemistry and Physics, Research Centre for Mediterranean Intensive Agrosystems and Agri-Food Biotechnology (CIAIMBITAL), University of Almeria, Agrifood Campus of International Excellence, ceiA3, E-04120 Almeria, Spain; ‡ Department of Analytical Chemistry, Faculty of Sciences, University of Granada, Campus Fuentenueva s/n, E-18071 Granada, Spain

**Keywords:** additives, HRMS, aromatic hydrocarbons derivatives, conservatives, greenhouse, SPME

## Abstract

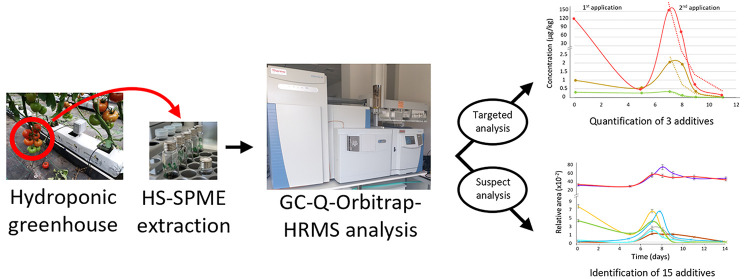

Additives present in plant protection products (PPPs) are normally not monitored after sample treatments. In this study, the fate of additives detected by targeted and nontargeted analysis in tomato samples treated with two PPPs was carried out. The study was carried out in a greenhouse for 12 days, in which two applications with each PPP were made. Compounds were extracted by applying a headspace solid phase microextraction (HS-SPME) and analyzed by gas chromatography coupled to high resolution mass spectrometry (GC-HRMS), performing targeted and suspect approaches. Three targeted and 15 nontargeted compounds were identified at concentration levels of up to 150 μg/kg. Compounds detected encompassed benzene, toluene, indene, and naphthalene derivatives, as well as conservatives and flavouring compounds. Most of them degraded in less than 7 days after the second application, following first-order kinetic. This study aims to reduce knowledge gaps regarding additives and their fate under real climatic conditions of greenhouses cultivations.

## 1 Introduction

The term “pesticide” is defined as any substance or mixtures of them employed to prevent, destruct, repel, or mitigate any pest.^1^ Pesticides are widely used in current agriculture to increase food production. However, these can produce severe health and environmental problems, including immunological and neurological disorders, cancer or genetic diseases, water and soil contamination, or the reduction of pollinators as bees.^2,3^ That is why international organizations have established strict regulations in foods, such as Codex Alimentarius,^4^ and the Directorate-General for Health and Food Safety (SANTE) in the European Union (EU),^5^ among others. These regulations are constantly improving, and they have helped to increase food safety.

Although active substances are controlled with routine analysis in all food matrices (including active substances and, in some cases, their metabolites), there are several compounds that could be found in plant protection products (PPPs), which have been scarcely studied. On one hand, some impurities that were incorporated into the formulation during the manufacturing process could be found. On the other hand, additives are added to improve the PPP characteristics, and these encompass different compounds such as solvents, stabilizers, emulsifiers, etc.,^6^ being classified as safeners, synergists, coformulants, and adjuvants.^7^ However, regulations regarding these compounds are scarce. For example, in Regulation (EU) No 547/2011, rules for PPP labeling are indicated, but additives are not included.^8^ Consequently, no or very few compounds are indicated in the PPP label together with active substances, and concentrations are not often indicated. There is only one regulation, Regulation 77 EC No 2021/383, which mentions additives unacceptable for inclusion in PPPs.^9^

The fact that additives are not controlled in PPPs does not mean that they cannot have health implications. Most of them are derivatives of benzene, toluene, or naphthalene, which are considered toxic if they are ingested or inhaled, being also toxic for aquatic life.^10^ Despite their possible health effects, they have barely been studied in real samples and published articles are mainly focused on their analysis in PPPs.^11−13^ For their analysis, two techniques have been employed, gas chromatography coupled to high resolution mass spectrometry (GC-HRMS) and liquid chromatography (LC) coupled to HRMS. When LC-HRMS is employed, a dilution of the PPPs and direct injection is commonly used, identifying 10 (6 of them confirmed)^14^ and 78 (9 of them confirmed)^15^ coformulants. LC was also used in the study of Balmer et al. to determine the dissipation of PPP additives under field conditions.^16^ Using LC separation, medium polarity to polar compounds could be detected. However, as most of the additives contained in PPP formulations are known to be nonpolar compounds, GC seems a better alternative. In this case, although dilution and direct injection has been also employed with GC-HRMS instruments,^10^ the volatile nature of most of them favors the use of HS. Indeed, in the study of Hergueta-Castillo et al.,^17^ where 21 additives were confirmed, a comparison between direct injection and HS was carried out, observing that the number of confirmed compounds was higher when HS was employed.

In all of these studies, the concentrations found for all the confirmed compounds were very high, above 200 g/L (>20% *w/v*) in some cases. These concentrations were even higher than the concentrations of active substances for some additives. However, as additive concentrations in treated samples are expected to be low, preconcentration techniques are needed. In this matter, in a previous published study, HS-solid phase microextraction (HS-SPME) was used to determine the fate of some additives after the application of PPPs on different food commodities in lab trials.^18^ In that study, 7 targeted coformulants were quantified in tomato samples at low concentrations, ranging from 1 to 70 μg/kg, showing the preconcentration capacity of this technique. Besides, a recently published article also employed HS-SPME and GC-HRMS to putatively identify 15 additives in tomato samples treated with the PPP Altacor.^19^

These two last articles and the article of Balmer et al.,^16^ constitute the only three published studies where additive fate was determined. However, only in the studies of Balmer et al.^16^ and Maldonado-Reina et al.,^19^ the dissipation was evaluated under field conditions. In those studies, it was observed that the amount of coformulants declines fast, although it was also indicated that for some cultivations, such as parsley and celery, certain additives remain at high concentrations 2 weeks after treatment, highlighting the need for control of these substances.

Due to the lack of information about the fate of additives after on-field treatments, this study aims to understand their behavior after the application of PPPs on tomato cultivation in a greenhouse. An approach consisting of HS-SPME coupled to GC-HRMS was applied to evaluate the presence of additives and their dissipation in samples treated with two different PPPs, flutriafol, and penconazole. Identification of the compounds was performed by targeted and nontargeted analysis (suspect approach) using a database with 164 compounds.

## 2 Materials and Methods

### 2.1 Materials and Instruments

Perfluorotributylamine from Thermo Fisher Scientific (Waltham, MD, USA) was used for GC-HRMS exact mass calibration. Styrene-*d*_8_ obtained from Merck (St. Louis, MO, USA) was employed as an internal standard (IS).

Multiple additives were acquired to search them in the applied samples: benzene, 1,2,4-trimethylbenzene, 1,3,5-trimethylbenzene, 4-isopropyltoluene, isopropylbenzene, *n*-butylbenzene, naphthalene, *n*-propylbenzene, *sec*-butylbenzene, styrene, *tert*-butylbenzene, and toluene from Dr. Ehrenstorfer (Augsburg, Germany) and 2,4-dimethylstyrene, 4-ethyltoluene, 1,3-diisopropylbenzene, pentamethylbenzene, biphenyl, 2-methylbiphenyl, 3-methylbiphenyl, 4-methylbiphenyl, and diphenylmethane from Merck (St. Louis, MO, USA). Stock solutions were prepared by exact weighing of 10 mg of solid or liquid substance and mixing it with 10 mL of ethyl acetate from Merck (to obtain a concentration of 1000 mg/L). Working solutions were prepared at 10 mg/L in ethyl acetate. All of the standard solutions were stored at −21 °C in the dark.

Additives were analyzed using a Thermo Fisher Scientific Trace 1310 GC system (Thermo Scientific, Thermo Fisher Scientific, San Jose, CA, USA). The instrument was equipped with an autosampler Triplus RSH. A Varian VF-5 ms column (30 m × 0.25 mm, 0.25 mm film thickness) supplied by Agilent Technologies (Santa Clara, CA, USA) was employed for compound separation. Separation was achieved by employing helium (99.9999%) as carrier gas at a flow rate of 1 mL/min. The GC system operated at an injector temperature of 250 °C in splitless mode. When the instrument was in standby, the injector was changed to a split ratio, which was set at 20:1. A flushing step at 100 mL/min was employed after each injection to avoid carryover between samples. Programming temperature was as follows: analysis started at 35 °C, and this temperature was kept constant for 10 min; then the temperature was increased until 75 °C in 8 min and then until 300 °C in 2 min; temperature was held 10 min, and the total run time was 30 min.

A Q-Exactive Orbitrap mass spectrometer from Thermo Fisher Scientific (Q-Exactive) was coupled to the chromatographic system. The analyzer operated using electron impact ionization mode (Energy = 70 eV), acquiring in full scan mode between 50 and 500 *m*/*z*. Transfer line and ionization source operated at 250 °C.

Data were processed using TraceFinder 4.0 (Thermo Fisher Scientific) for targeted and suspect analysis.

### 2.2 PPPs Application and Sample Treatment

PPPs were applied over a tomato cultivation located in Almeria (Spain) in an agricultural field (greenhouse) using a hydroponic system. PPPs were not previously applied over this cultivation or over the previous one. Greenhouse area was about 727.2 m^2^, in which 204 tomato plants were cultivated. Plants were divided into three blocks (52 plants for each block). Blocks were separated between each other by 2 lines of plants (48 plants), which were not applied. One block was sprayed with Impact Evo 12.5% (active substance flutriafol) and another with Topas 20% (active substance penconazole). In both blocks, two applications were sprayed over the plants at the recommended dose (0.075% v/v for Impact Evo and 0.015% v/v for Topas), the second application a week after the first one. The third block was used as blank samples, ensuring that they were not contaminated by nearby applications. These samples were used to perform calibration curves. Every block was divided into three plots, each of them being a replicate.

Approximately 0.5 kg of tomato was collected, by triplicate, at 2 h and 5 days after first application and 2 h and 1, 2, 4, and 7 days after second application. Table 1 shows the characteristic parameters of the greenhouse experiment. All samples were collected at the same value (60) according the Biologische Bundesanstalt, Bundessortenamt and Chemische Industrie scale (BBCH). Tomato samples were crushed, stemless, and without washing step, according to current national legislation.^20^ Then 5 g of crushed sample was weighed in a SPME vial and 25 μL of a styrene-*d*_8_ (Dr. Ehrenstorfer) solution (10 mg/L) was added to all samples to normalize the signals. The samples were homogenized in vortex, and they were submitted to the HS-SPME extraction. The method was previously developed, concluding that HS-SPME provides better analytical performance than other as HS.^18^ A polydimethylsiloxane (PDMS) SPME fiber purchased by Merck was used. Some of the set parameters were: 1 min of incubation time at 70 °C, 30 min of extraction with agitation each 10 s time, and 3 min of desorption time.

**Table 1:** Parameters of Greenhouse Experiment

study location	Retamar, Almeria, Spain
orientation	east–west
area	727.2 m^2^
working field	204 tomato plants
agricultural model	passive climate control systems with 4% ventilation and side windows (12.9% ventilation)
cultivation system	hydroponic crop
irrigation water	0.6–3.0 dS/m
application dose	0.090 L/h
air temperature (greenhouse indoor)	16.5 °C
binomial plant name	*Solanum lycopersicum* L. (tomato)
plant stage at pesticide application	bloom
Biologische Bundesanstalt, Bundessortenamt and Chemische Industrie scale	60

## 3 Results and Discussion

### 3.1 Targeted Analysis

A targeted study was carried out to identify the most frequently identified additives in the incurred samples. The 21 additives already detected in previous studies with similar PPP formulations were acquired and searched in tomato samples collected from the greenhouse after the first and second application.^10,17−19^ However, only 3 of them were detected in the treated samples, for both treatments, 1,2,4-trimethylbenzene, pentamethylbenzene and biphenyl.1,2,4-trimethylbenzene and biphenyl had been detected in Topas PPP at a concentration of 1.91 and 0.01 g/L, respectively,^17^ and 1,2,4-trimethylbenzene and pentamethylbenzene were detected when Altacor PPP was sprayed over tomato cultivation, but they were neither confirmed nor quantified.^19^ In this study, they were detected with concentrations for both PPPs treatments between 0.42 and 160.14 μg/kg. These compounds are derived from benzene, and they could exhibit irritated, toxic, and narcotic effects.^10^ In the case of 1,2,4-trimethylbenzene, the compound found at highest concentration, it is considered a neurotoxic compound and, although the found concentrations were low, these compounds can cause systemic effects due to long-term exposures, as it was indicated in the safety sheet for aromatic hydrocarbon compounds.^21^

**Figure 1:**
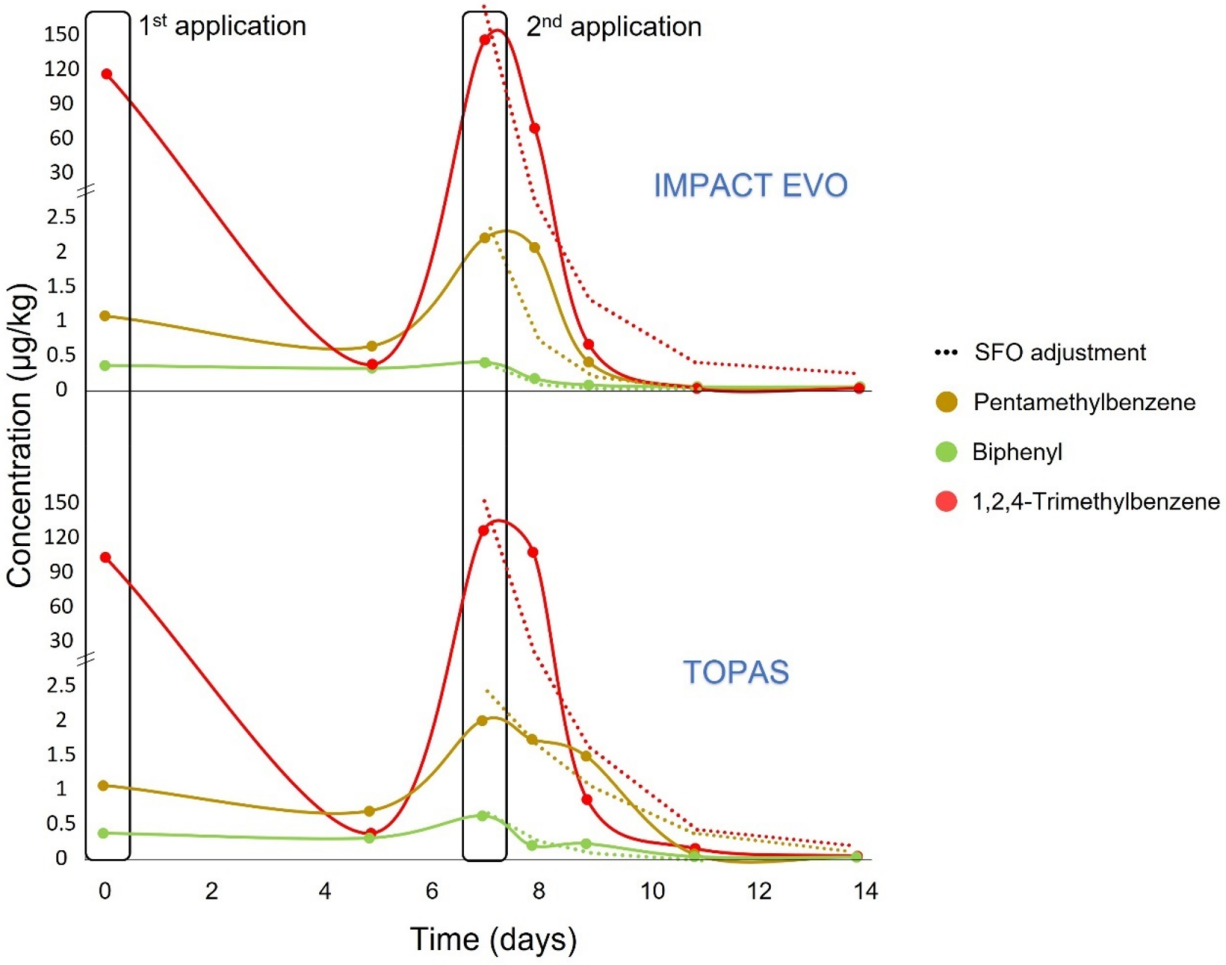
Dissipation curves of targeted detected compounds in both applications.

The behavior of these compounds was studied throughout the monitoring period. As it can be seen in Figure 1, their concentrations increased after each application and then they decreased quickly. Dissipation curves were adjusted to a single first-order (SFO) adjustment with *R*^2^ higher than 0.90 for the three compounds in the two PPPs evaluated. Thus, 11 days after the first application, the compounds were not detectable. In fact, in all cases, the half-time (DT_50_) was less than 2 days, and the time it took for the initial concentrations to decrease by 90% (DT_90_) was less than 7 days, as shown in Table 2. While the kinetic parameters for 1,2,4-trimethylbenzene appear to be independent of the PPP applied, differences are observed for the other two substances, particularly pentamethylbenzene, despite being applied in the same matrix. Therefore, further studies on different samples are needed to determine whether other components of the PPP, such as the active ingredient or other additives not identified by the targeted method, can affect the degradation kinetics of these substances.

**Table 2:** Biphasic Kinetic Model Parameters and Half Life Times (DT_50_) of Target Compounds Identified

	Impact Evo			Topas		
matrix	1,2,4-trimethylbenzene	pentamethylbenzene	biphenyl	1,2,4-trimethylbenzene	pentamethylbenzene	biphenyl
[initial] (μg/kg)	160.14	2.72	0.42	158.70	2.50	0.74
*k* (h^–1^)	0.69	1.58	2.25	0.58	0.36	1.16
DT_50_(days)	1.00	0.44	0.31	1.20	1.93	0.60
DT_90_(days)	3.34	1.45	1.03	3.98	6.41	1.99
*R*^2^	0.95	0.90	0.91	0.95	0.97	0.93
retention time (min)	18.42	20.56	20.49	18.42	20.56	20.49

Comparison of these results with those obtained from PPPs^10,17^ reveals that these three substances are commonly detected in a significant proportion of PPPs or in samples where they are applied. For example, pentamethylbenzene was identified as one of the most identified additives in PPPs, often at higher concentrations.^10^ The confirmed compounds were already detected in a laboratory trial, where PPP Mitrus (active substance myclobutanil) was applied to tomato and grape samples already collected.^18^ In that previous study, the compounds had a slightly different behavior: their concentrations decreased after application, then increased, reaching the maximum on day 2, and finally decreased again, probably due to the different ambient conditions. However, like in greenhouse treatment, after 12 days, the compounds were totally degraded.

The sole published study examining the dissipation of additives in field samples monitored 4 specific coformulants resulting from the utilization of three distinct PPPs across a diverse selection of fruits and vegetables.^16^ They found that in most vegetables, coformulants disappear after a few days (15% remain 3 days after application in most cultivations). However, dissipation of anionic coformulants was affected by a rainfall event. Thus, the half-life could be longer without this wash-off process. Organic additives had a similar behavior, but they were more influenced by a volatilization process (decline was produced before the rain event). When the nature of the additives identified in the current study is taken into account, volatilization also seems to be the main dissipation route. Furthermore, because cultivation was carried out with a hydroponic crop, wash-off cannot affect the dissipation of these additives.

The applied PPPs have both withdrawal periods of 3 days after the last application. Thus, after 3 days, the targeted additives were not identified, considering the risk of consuming food treated with these PPPs is low in terms of the presence of additives.

### 3.2 Suspect Analysis

In addition to the targeted compounds monitored in the previous section, and bearing in mind that more additives could be present in the PPPs, a large list of additives was included in a homemade database, and they were searched in the treated samples. As identification criteria, a mass error lower than 5 ppm and the detection of at least two ions were required. The database includes 164 compounds, which were previously detected in other on-lab or field trials or described by EFSA as coformulants used in representative PPP formulations.^22^ This database can be found in Supporting Information (SI), Table S1 as a directly exportable CSV to TraceFinder software. It should be considered that some compounds are isomers, and thus when one of them was identified, it is not possible to assign the signal to one particular compound, unless standards were acquired.

**Figure 2:**
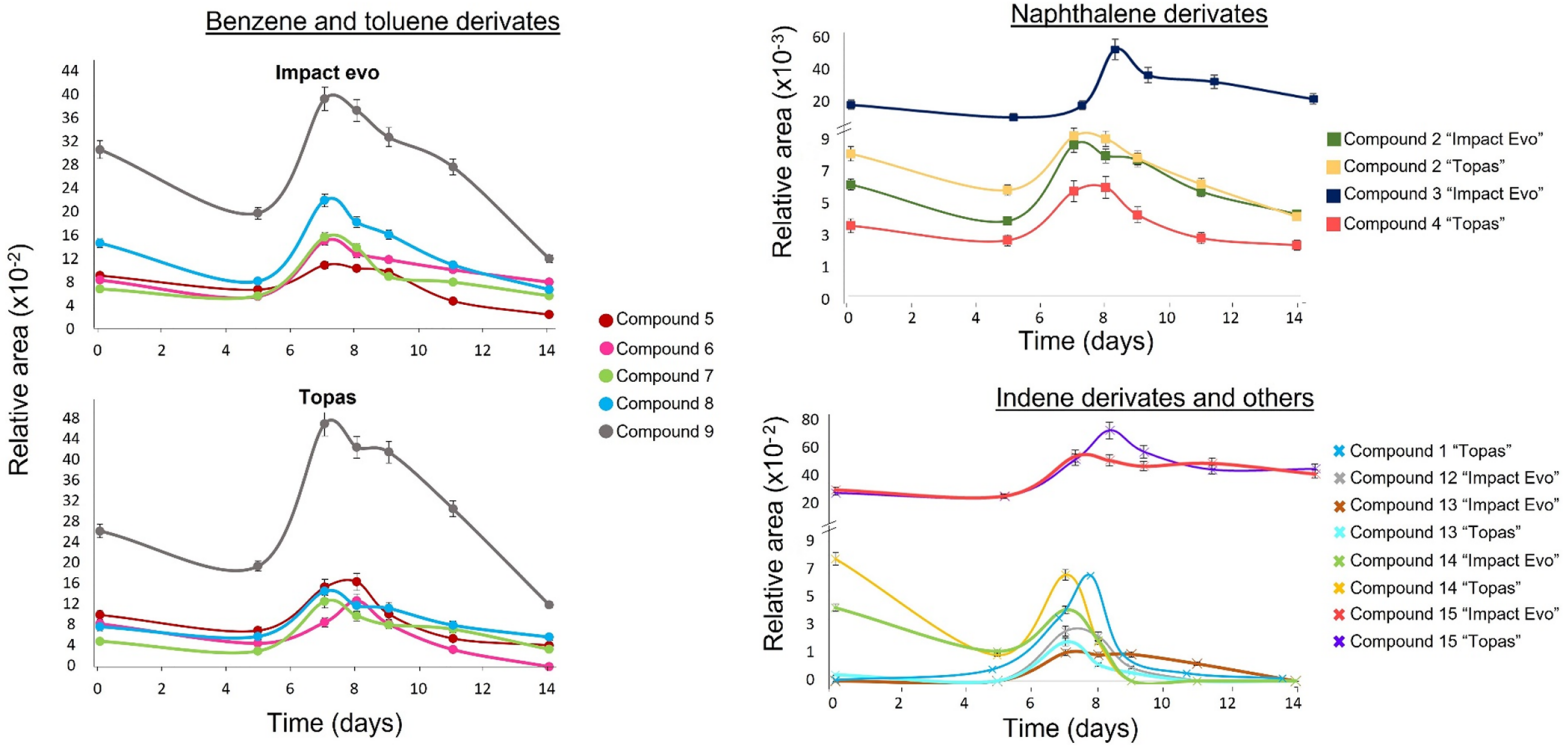
Dissipation curves of nontargeted identified compounds. Compound code: **1** = methylparaben; **2** = allylnaphthalene; **3** = 1-methylnaphthalene; **4** = trimethylnaphthalene; **5** = ethylbenzene; **6** = 1,4-diethylbenzene/1-methyl-3-propylbenzene/ethylxylene/tetramethylbenzene/isopropyltoluene/α-methylstyrene; **7** = 4-*tert*-butyl-*o*-xylene; **8** = oxyphenalon/4-methyl-2-phenyl-1,3-dioxolane; **9** = 2,4,6-trimethylstyrene; **10** = butylated hydroxytoluene; **11** = 3,4-dimethylbiphenyl; **12** = indane; **13** = 1,2-dimethylindane; **14** = linalool; **15** = *d*-limonene

Fifteen compounds were tentatively identified in the tomato samples, none of them being detected in the blank samples (Table 3 and Figure 2). As there were no analytical standards for these compounds, they were expressed as relative areas using the internal standard described above. Most of them were found in both treatments, except methylparaben, trimethylnaphthalene, and 3,4′-dimethyl-1,1′-biphenyl, which were only detected in Topas treated samples, and 1-methylnaphthalene, butylated hydroxytoluene and ethylxylene/isopropyltoluene/propyltoluene/*tert*-butylbenzene/tetramethylbenzene/α-methylstyrene/4-ethyl-*m*-xylene, which were only detected in Impact Evo treated samples. Therefore, although the same compounds could be detected in both PPPs, there are specific additives that are only identified in one PPP, highlighting the need to monitor this type of products in both PPPs and applied samples. Annotated compounds encompassed different compound families as parabens (methylparaben), used as antimicrobials and preservatives,^23^ naphthalene, benzene, toluene, and indene derivatives (compounds 2–13), mentioned in the safety sheets as “aromatic hydrocarbons and naphthalene derivatives compounds”, and other compounds use as flavor (linalool and limonene).

**Table 3:** Estimated Concentrations for Nontargeted Compounds Found in the Treated Samples

			estimated concentrations (μg/kg)					
			first application	second application				
compd family	compd^a^	retention time (min)	2 h	2 h	1 day	2 days	4 days	7 days
Impact Evo								
naphthalene	**2**	21.08	0.39	0.53	0.49	0.48	0.37	0.29
	**3**	20.82	1.89	1.86	4.16	3.10	2.83	2.12
benzene and toluene	**5**	21.44	17.28	20.73	19.66	18.38	8.97	4.49
	**6**	21.46	15.83	28.71	24.51	22.54	19.23	15.16
	**7**	21.53	12.88	29.88	26.40	16.96	15.14	10.67
	**8**	21.41	27.94	41.92	34.77	30.74	20.83	12.71
	**9**	21.49	58.56	75.19	71.38	62.65	52.89	22.79
	**10**	20.70	1.03	1.86	1.08	0.66	0.21	
indene	**12**	21.35		9.48	8.46	2.59		
	**13**	21.40		5.39	4.99	5.08	3.29	
Topas								
paraben	**1**	19.96						
naphthalene	**2**	21.08	0.50	0.56	0.55	0.49	0.39	0.28
	**4**	21.26	0.25	0.37	0.38	0.29	0.20	0.18
benzene and toluene	**5**	21.44	6.79	10.43	11.13	6.91	3.71	2.81
	**6**	21.46	5.64	5.82	8.66	5.60	2.25	
	**7**	21.53	3.34	8.57	6.68	5.51	4.92	2.29
	**8**	21.41	5.26	9.91	8.04	7.66	5.46	3.89
	**9**	21.49	17.79	31.87	28.79	28.16	20.73	8.10
	**11**	21.31	0.29	0.68	0.46	0.29	0.18	
indene	**13**	21.40	0.37	2.30	0.98	0.49		

a Compound code: **1** = methylparaben; **2** = allylnaphthalene; **3** = 1-methylnaphthalene; **4** = trimethylnaphthalene; **5** = ethylbenzene; **6** = 1,4-diethylbenzene/1-methyl-3-propylbenzene/ethylxylene/tetramethylbenzene/Isopropyltoluene/α-methylstyrene; **7** = 4-*tert*-butyl-*o*-xylene; **8** = oxyphenalon/4-methyl-2-phenyl-1,3-dioxolane; **9** = 2,4,6-trimethylstyrene; **10** = butylated hydroxytoluene; **11**= 3,4-dimethylbiphenyl; **12** = indane; **13** = 1,2-dimethylindane.

The relative areas of the detected compounds were different depending on their compound family (Figure 2). While benzene derivatives and limonene reached high relative areas (up to 0.48), naphthalene and indene derivatives, methylparaben, and linalool reached lower relative areas (up to 0.09). Their behavior along days was also related to their compound family (Figure 2). A semiquantification step was carried out, and their concentrations were calculated with those matrix match calibration curves of some of the targeted available standards with similar characteristics (naphthalene derivatives with naphthalene calibration curve and benzene and toluene derivatives with benzene calibration curve) (Table 3).

The concentrations of the benzene-derived compounds were up to 75 μg/kg. These concentrations were lower than those obtained in the study of Balmer et al., where some compounds had concentrations up to 10 mg/kg.^16^ Moreover, they degraded quickly (between 47 and 100% was dissipated in 11 days). On the other hand, naphthalene derivatives seem to be more stable (46–53% degraded in 7 days after a second application). However, the estimated concentrations were very low for these compounds (up to 4 μg/kg). Finally, indene derivatives (concentrations lower than 10 μg/kg), linalool, and methylparaben degraded even faster than benzene derivatives (100% in 7 days or less) and 30–45% of limonene remains after 11 days. Like targeted compounds, the detected compounds are considered to be volatile compounds. Therefore, volatilization seems to be the main dissipation route. Indeed, dissipation differences between families could be related to their volatility: benzene derivatives have lower enthalpy of vaporization (Δ*H*_vap_) than naphthalene derivatives, e.g., benzene has a Δ*H*_vap_ = 33.83 kJ/mol, while naphthalene has a Δ*H*_vap_ = 45.13 kJ/mol.^24,25^ This could be the reason that benzene derivatives dissipated faster than naphthalene derivatives.

Results were statically compared by applying a *t* test (assuming equal sample variances) between applications (SI, Table S2). The concentration of compounds simultaneously detected in samples treated with both PPPs were significantly higher (*p*-value <0.05) in Impact Evo with respect to Topas, except for allylnaphthalene which was similar in both treatments (*p*-value = 0.64). This may be because the application dose for Impact Evo (0.075% v/v) is higher than that for Topas (0.015% v/v). Besides, if their initial (after the second application) and final concentrations were considered, results shown that differences were maintained during the dissipation process (*p*-values <0.001 for the initial and final conditions). This can be explained because, although initial concentrations were different between treatments, degradation is independent of concentrations, being the differences between them constant.

According to these results, the concentrations of compounds supposed to cause health outcomes, such as benzene, toluene, and indene, decreased in a few days. Therefore, if withdrawal periods are fulfilled, they should not be considered harmful if they are ingested through the diet (when tomatoes are treated with PPPs for example). Regarding naphthalene compounds, although they persist in the fruit for a longer period of time, their initial concentrations were lower than those of the benzene derivatives. On the other hand, linalool and limonene are not considered harmful, being naturally present in many plants. Finally, although methyl paraben is known to be an endocrine disruptor chemical, it is also present in a wide variety of dairy products at higher concentrations and it has a low toxicity and lower endocrine disrupting activity compared with other parabens.^23,26^

## Conclusions

In conclusion, this study carried out the monitoring during 14 days of volatile additives after the application of tomatoes with two PPP in a greenhouse. Some compounds previously detected in similar PPPs have also been identified in treated samples, exhibiting first-order degradation kinetics with half-lives lower than 2 days, suggesting that they degrade before harvest. However, further studies are needed to assess their degradation under other matrices and conditions. Additionally, a suspect analysis revealed more compounds present at lower concentrations but with greater stability. This indicates the need to monitor them in routine studies to evaluate the safety of fruits and vegetables treated with PPPs, taking into account that these substances along with the active ingredient have the potential to cause toxic effects on health. This work comprises the first one that explores the fate of additives after greenhouse application by combining targeted and suspect analyses.

## Supporting Information Available

Nontargeted database used during suspect analysis and the *t* test statistical analysis performed for the common suspect compounds (XLSX)
